# HECTAR: A method to predict subcellular targeting in heterokonts

**DOI:** 10.1186/1471-2105-9-393

**Published:** 2008-09-23

**Authors:** Bernhard Gschloessl, Yann Guermeur, J Mark Cock

**Affiliations:** 1 UPMC Univ Paris 6, UMR 7139 Végétaux marins et Biomolécules, Station Biologique, F 29682, Roscoff, France; 2 CNRS, UMR 7139 Végétaux marins et Biomolécules, Station Biologique, F 29682, Roscoff, France; 3 LORIA-CNRS, Campus Scientifique, BP 239, 54506 Vandœuvre-lès-Nancy cedex, France

## Abstract

**Background:**

The heterokonts are a particularly interesting group of eukaryotic organisms; they include many key species of planktonic and coastal algae and several important pathogens. To understand the biology of these organisms, it is necessary to be able to predict the subcellular localisation of their proteins but this is not straightforward, particularly in photosynthetic heterokonts which possess a complex chloroplast, acquired as the result of a secondary endosymbiosis. This is because the bipartite target peptides that deliver proteins to these chloroplasts can be easily confused with the signal peptides of secreted proteins, causing currently available algorithms to make erroneous predictions. HECTAR, a subcellular targeting prediction method which takes into account the specific properties of heterokont proteins, has been developed to address this problem.

**Results:**

HECTAR is a statistical prediction method designed to assign proteins to five different categories of subcellular targeting: Signal peptides, type II signal anchors, chloroplast transit peptides, mitochondrion transit peptides and proteins which do not possess any N-terminal target peptide. The recognition rate of HECTAR is 96.3%, with Matthews correlation coefficients ranging from 0.67 to 0.95. The method is based on a hierarchical architecture which implements the divide and conquer approach to identify the different possible target peptides one at a time. At each node of the hierarchy, the most relevant outputs of various existing subcellular prediction methods are combined by a Support Vector Machine.

**Conclusion:**

The HECTAR method is able to predict the subcellular localisation of heterokont proteins with high accuracy. It also efficiently predicts the subcellular localisation of proteins from cryptophytes, a group that is phylogenetically close to the heterokonts. A variant of HECTAR, called HECTAR^*SEC*^, can be used to identify signal peptide and type II signal anchor sequences in proteins from any eukaryotic organism. Both HECTAR and HECTAR^*SEC *^are available as a web application at the following address: .

## Background

Many cellular processes depend on proteins being targeted to specific subcellular localisations. As a result, information about the subcellular localisation of a protein can provide important insights into its function. Conversely, knowledge about which proteins are targeted to a specific subcellular localisation can lead to a better understanding of the functions of a particular compartment of the cell. This can be particularly interesting in an evolutionary context. For example, mitochondria and chloroplasts have evolved from being enslaved organisms, engulfed by a host cell, to become specialised cellular compartments integrated into the functioning of the host cell [1,2]. Identification of proteins that are targeted to these organelles can provide clues as to how these organelles evolved.

A large variety of methods have been developed to predict the subcellular localisation of nuclear encoded proteins. Pattern recognition methods which have been most favoured are Hidden Markov Models (HMMs) [3-5], Neural Networks (NNs) [6-8] and Support Vector Machines (SVMs) [9-12]. Bayesian methods [13] and fuzzy k-nearest neighbour algorithms [14], linear discriminant analysis (LDA) [15], position weight matrices [16] and rule based systems [17] have also been proposed. All subcellular localisation prediction methods use various approaches to interpret the intrinsic information present in protein sequences. The composition of amino acids within the polypeptide sequence or the composition of peptide sequences of fixed length (n-gram) [13,14], sequence profiles [10], physio-chemical parameters like hydrophobicity, charged residues and isoelectric points as well as details about the secondary structure like amphiphilic alpha helices, membrane regions and the orientation of N- and C-terminal ends are taken into account [3,5,15,17].

The majority of subcellular prediction programs search for N-terminal targeting peptides since these sequences are common in proteomes [18-20].

**Figure 1:**
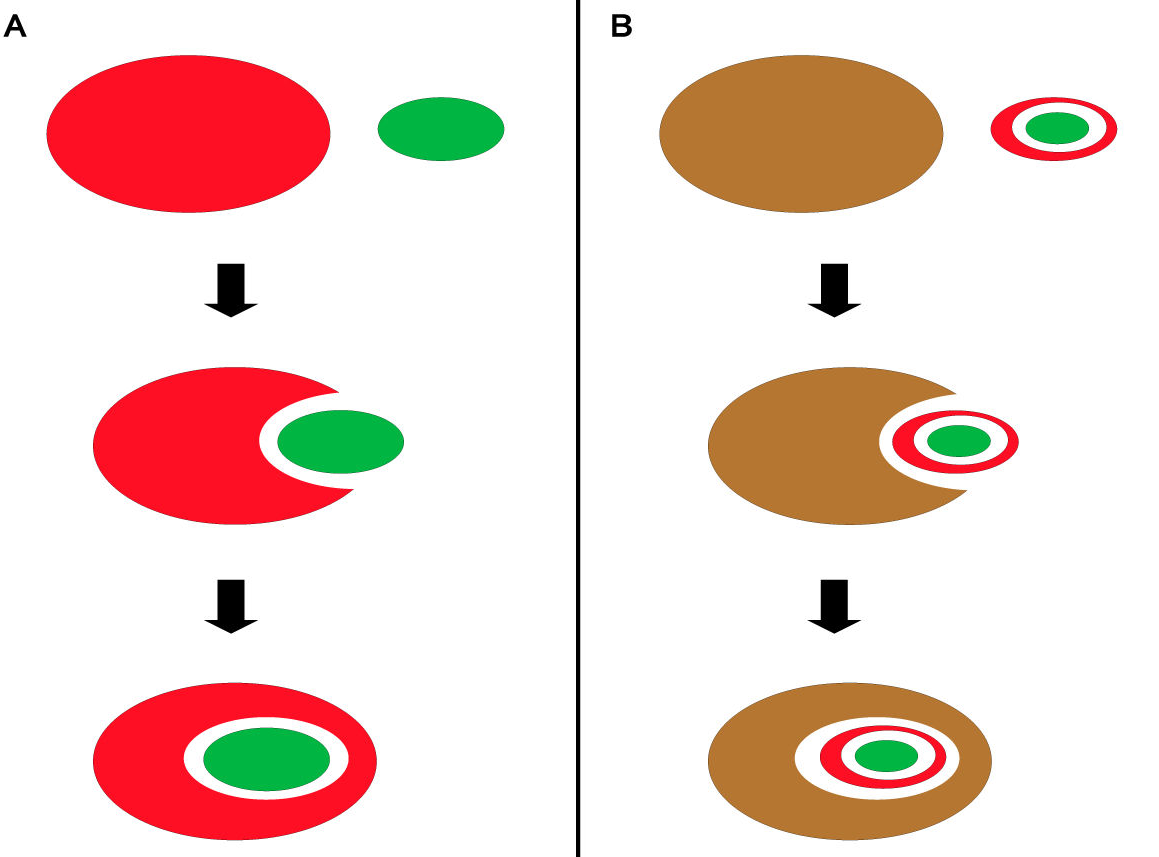
**Primary and secondary endosymbiosis**. **part A: **Primary endosymbiosis is proposed to have involved the capture of a cyanobacterium (green elipse) by a eukaryotic heterotroph (red elipse). The cyanobacterium would then have been modified during evolution to give rise to a plastid with two surrounding membranes. **part B: **The secondary endosymbiotic event that gave rise to the heterokonts is proposed to have involved the engulfment of a red algae with a chloroplast (green elipse inside a red elipse) by a eukaryotic heterotroph (brown elipse). The red alga would have become the heterokont plastid with four surrounding membranes.

**Figure 2:**
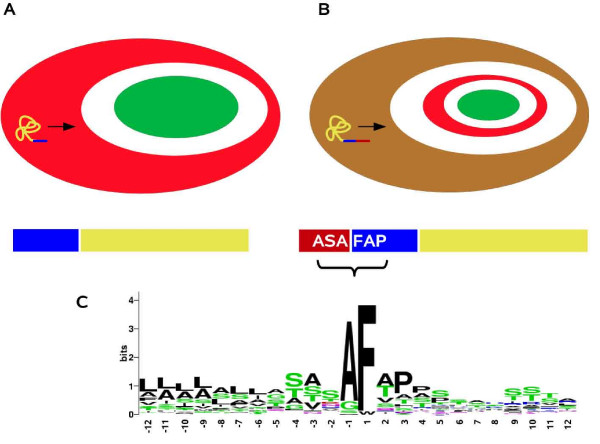
**Comparison of the chloroplast target peptides of red algae and heterokonts**. **part A: **To be imported into a red algal chloroplast, proteins require only a single N-terminal transit peptide (blue bar). The transit peptide is cleaved when the protein passes through the outer chloroplast membrane. The mature protein (yellow) is then transferred into the chloroplast. **part B: **In heterokonts, proteins which are targeted to the chloroplast possess a bipartite target peptide which is made up of an N-terminal signal peptide (red) followed by a chloroplast transit peptide (blue). The signal peptide is cleaved when the protein passes through the outermost of the four heterokont chloroplast membranes. This places the transit peptide at the new N-terminus of the protein where it can mediate transfer across the two innermost membranes. The transit peptide is cleaved during this second step of the transfer. **part C: **Sequence logo of the conserved ASAFAP motif surrounding the predicted signal peptide cleavage site based on 55 chloroplast targeted proteins from heterokonts. The logo was built with WebLogo [53] and is based on manually improved alignments of the sequence neighbouring the predicted cleavage site.

The heterokonts are a diverse evolutionary group that includes diatoms, brown algae, and oomycete plant pathogens such as potato late blight [21]. The plastids of the photosynthetic members of this group are thought to be derived from a secondary endosymbiotic event involving a red alga and a eukaryotic heterotroph (Fig. 1). The enslavement of the endosymbiont involved alterations to its structure and most of the endosymbiont's genes were transfered to the host nucleus or lost [22,23]. As a result, present day heterokonts possess plastids with a complex structure. These organelles are surrounded by four concentric membranes and the nuclear-encoded proteins that function in these plastids have to be transported into the organelle through these four membranes. This is mediated by bipartite, N-terminal targeting sequences consisting of a leading signal peptide followed by a chloroplast transit peptide (Fig. 2). Kilian and Kroth [24] have identified a conserved motif (ASAFAP) at the cleavage site of the signal peptide in heterokont plastid targeted proteins. This motif includes the alanine (A) residues at positions -1 and -3 relative to the cleavage site that are commonly found in all signal peptides [25]. The phenylalanine (F) residue just after the cleavage site is highly conserved, it occurs in almost all heterokont chloroplast targeted proteins, being only rarely replaced by tryptophan, tyrosine or leucine [26]. The ASAFAP motif was shown to be essential for import into the plastid in experiments that involved transforming the diatom *Phaeodactylum tricornutum *with constructs encoding modified plastid target peptides fused to green fluorescent proteins (GFP) [24,26].

We show here that the unusual structure of heterokont plastid targeting sequences causes problems for currently available subcellular prediction methods that have been designed to predict the subcellular localisations of proteins from other eukaryotic groups such as animals, fungi and green plants. To overcome this difficulty, we have created a new method that is specifically designed to predict the subcellular localisation of heterokont proteins.

## Results and discussion

### Shortcomings of existing prediction tools when applied to heterokont chloroplast targeted proteins

To determine how well existing subcellular localisation prediction methods perform on chloroplast targeted proteins from heterokonts, we submitted 55 experimentally verified sequences (see Methods for details) to four widely used subcellular prediction methods: TargetP [8] (v. 1.1), Predotar [6] (v. 1.03), PredSL [27] (v. 2005) and iPsort [17] (v. 2002). These methods assign proteins to one of four different subcellular localisations: the secretory pathway, based on the presence of a signal peptide, the chloroplast or the mitochondrion, based on the presence of the respective transit peptides, or to none of these three localisations, based on the absence of a detectable N-terminal targeting sequence. As expected, these prediction methods erroneously assigned a significant proportion of the chloroplast proteins to the secretory pathway (see Table 1). In addition, Predotar failed to detect the presence of a target peptide in a significant number of proteins, and some proteins were falsely predicted as being targeted to the mitochondrion by three of the four methods. In contrast, we were able to show that the signal peptide component of the bipartite chloroplast targeting sequences was efficiently recognised by methods that have been developed to specifically distinguish proteins with signal peptides (designed for the secretory pathway) from non-secreted proteins. The four methods tested, Phobius [3] (v. 1.01), PrediSi [16] (v. 2003) and both the Neural Network and Hidden Markov Model versions of SignalP [7] (v. 3.0) (SignalP_NN and SignalP_HMM), identified the signal peptide component of 55, 48, 53 and 55 of the 55 heterokont chloroplast proteins, respectively. Based on this result, we decided to create a method that would be able to efficiently recognise the bipartite chloroplast targeting sequences of heterokonts by searching for each of the two components of this structure in a step by step manner.

**Table 1:** Analysis of heterokont chloroplast targeted proteins using four currently existing subcellular localisation predicting algorithms.

	**Secretory pathway**	**Chloroplast**	**Mitochondrion**	**No target peptide**
TargetP	20	25	6	4
Predotar	35	0	0	20
PredSL	25	26	3	1
iPsort	40	6	9	0

The subcellular localisation predicting algorithms TargetP, Predotar, PredSL and iPsort were used to analyse 55 heterokont chloroplast targeted proteins. For TargetP we declared a protein as possessing no N-terminal target peptide if the highest category support probability was less than 0.32.

### A hierarchical procedure to recognise heterokont chloroplast targeting sequences

**Figure 3:**
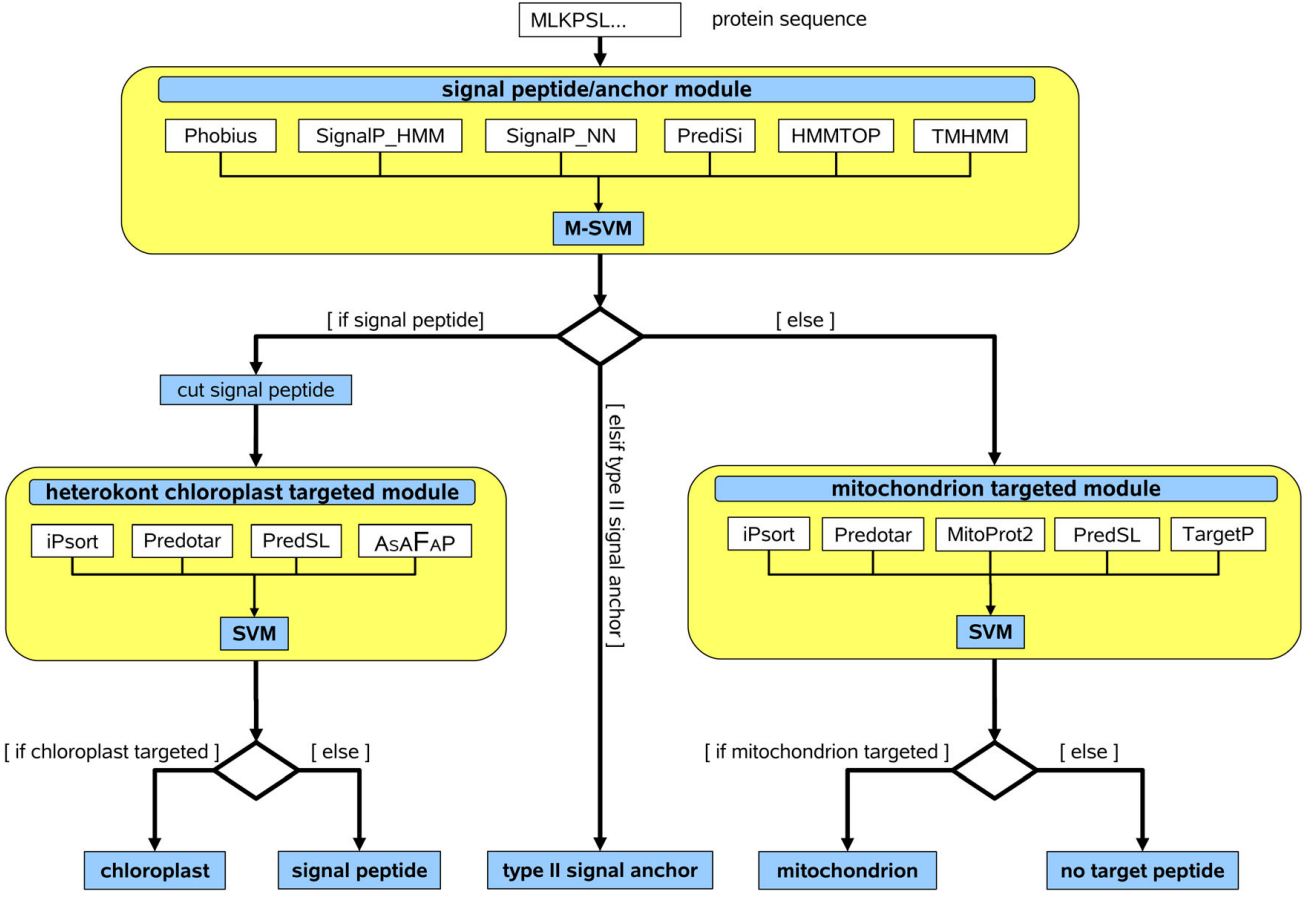
**Hierarchical architecture of HECTAR**. Five categories of subcellar targeting can be predicted by the HECTAR method: signal peptides, type II signal anchors, chloroplast transit peptides, mitochondrion transit peptides and proteins with no detectable N-terminal target peptide. Each decision module (yellow boxes) runs several selected methods (white boxes) to detect specific target peptides. Selected outputs from these methods are then submitted to a SVM which combines these predictors to determine whether a particular target peptide is present in the sequence being analysed. Protein sequences are first analysed by the "signal peptide/anchor" module where a multi-class SVM determines whether a signal peptide or a type II signal anchor is present. If a signal peptide is detected, this sequence is removed from the N-terminal end of the protein sequence and the modified sequence is analysed by the "chloroplast targeted" module which determines whether the signal peptide is followed by a chloroplast transit peptide. In this module the result of a search for the ASAFAP motif is included in the decision process. If a chloroplast transit peptide is present the protein is classified as chloroplastic, otherwise it is classified as having either a signal peptide or a type II signal anchor. When the "signal peptide/anchor" module did not predict either a signal peptide or a type II signal anchor, the protein sequence is analysed by the "mitochondrion targeted" module to determine whether a potential mitochondrion target peptide is present. If a mitochondrion target peptide is found, the protein is assigned as being targeted to the mitochondrion, otherwise it is classified as having no N-terminal target peptide.

The HEterokont subCellular localisation TARgeting method (HECTAR) has a hierarchical architecture consisting of three decision modules (Fig. 3). Each module is dedicated to the identification of one or two specific N-terminal target peptides. Altogether, four target peptides can be predicted: signal peptides, type II signal anchors, chloroplast transit peptides and mitochondrion transit peptides. The absence of any detectable target peptide represents a fifth category. The decision module at the root of HECTAR (the "signal peptide/anchor" module) differs from the two other modules in that it discriminates between three categories: N-terminal signal peptides, type II signal anchors and proteins without either of these target peptides. Type II signal anchors resemble secretory signal peptides but have a longer N-terminal hydrophobic region which is able to span the membrane. Type II signal anchors are not cleaved. Their function is to anchor proteins in the membrane. The N-terminus of a type II signal anchor protein is located in the cytoplasm (N_*in*_) whilst the C-terminus projects into the lumen of the endoplasmic reticulum or is on the outside of the cell (C_*out*_) [28,29].

The first decision module of HECTAR identifies signal peptides and type II signal anchors by combining the predictions of Phobius, PrediSi, SignalP_NN and SignalP_HMM. HMMTOP [30] (v. 2.1) and TMHMM [4] (v. 2.0c) can detect the long hydrophobic regions which characterise signal anchors and were therefore also incorporated into the decision process. If HECTAR predicts a type II signal anchor, the category for this protein has been found. Otherwise, if a signal peptide is detected, the protein is further analysed to determine whether the signal peptide is a component of a chloroplast targeting sequence. To do this, HECTAR first searches for the ASAFAP motif around the predicted signal peptide cleavage site (see Methods for details). HECTAR then cleaves the signal peptide based on the signal peptide cleavage site predicted by Phobius, PrediSi, SignalP_NN and SignalP_HMM. The truncated protein sequence, lacking the signal peptide, is then analysed by the "chloroplast targeted" module which determines whether a chloroplast transit peptide is present at what is now the N-terminal end of the protein sequence. The chloroplast targeted module combines the calculated score for the appearance of the ASAFAP motif with the output of the plant versions of Predotar, iPsort and PredSL. Initially, we also considered integrating ChloroP [31] (v. 1.1) into this module. However, this method predicted a chloroplast localisation for many secreted proteins after the leading signal peptide had been removed (data not shown). If a chloroplast transit peptide is detected by the "chloroplast targeted" module, the protein is classified as being chloroplastic, otherwise it is labelled as being part of the secreted pathway. Going back to the root of the hierarchy, if no signal peptide/anchor is detected at this level, the protein sequence is fed to the module that identifies mitochondrion targeted proteins. This module combines the predictions of MitoProt2 [15] (v. 1.101) and the non-plant versions of TargetP, Predotar, iPsort and PredSL. If a mitochondrion targeting sequence is detected, the protein is classified as mitochondrial, otherwise, it is assigned to the default category "no N-terminal target peptide", indicating that it is located in the cytosol or subcellular compartments where protein targeting does not require a N-terminal target peptide.

### Use of support vector machines as an ensemble method for detecting target peptides

Since the early sixties, and more precisely since the work of Bates and Granger [32,33], model combination has proved to be an efficient alternative to model selection for a wide range of statistical inference problems. Theory in the field has made rapid strides, first in the framework of regression, and more recently in discrimination. The success of methods such as *bagging *[34] and *boosting *[35] has highlighted the usefulness of implementing *large margin *ensemble methods to improve the performance of *weak classifiers*. As mentioned above, programs that predict the subcellular localisations of proteins are based on different principles, and therefore provide complementary information (i.e., their errors are not too correlated). This is why HECTAR was designed to combine the output of selected prediction methods at each node of the hierarchy (Fig. 3). We chose to use SVMs for this task. This choice was based on the usefulness of large margin models to combine classifiers, and on the fact that SVMs have already proved very efficient to combine prediction methods in the field of protein sequence processing (see for instance [36,37]). The "signal peptide/anchor" module discriminates between three categories. For this module, the multi-class SVM (M-SVM) of Weston and Watkins was used [38]. The kernel of the SVMs incorporated in the "signal peptide/anchor" module and the "mitochondrion targeted" module is a Radial Basis Function (RBF), the one for the "chloroplast targeted" module is linear. This choice was based on preliminary test results (data not shown). For each of the three SVMs, an optimal subset of the outputs of the base classifiers was selected to constitute the set of predictors. This selection was performed based on the biological significance of the outputs.

### Assessment of the prediction accuracy of HECTAR

A set of reference proteins was established for each of the five categories of subcellular targeting predicted by HECTAR (see Methods for details). The entire set comprised 441 secretory path proteins, 11 type II signal anchor proteins, 55 heterokont chloroplast targeted proteins, 128 mitochondrion targeted proteins and 1423 nuclear/cytosolic proteins. A five-fold cross-validation procedure was applied to this set to assess the prediction accuracy according to two criteria: the recognition rate and the Pearson's/Matthews' correlation coefficients (MCC) [39].

In our data set, the proportions of type II signal anchor proteins, chloroplast targeted proteins and mitochondrion targeted proteins are 0.5%, 2.7% and 6.2% respectively. This implies that the training sets of the three SVMs are highly unbalanced, which could *a priori *have a negative influence on the accuracy of the prediction. Based on this observation, we decided to assess the usefulness of reducing the number of negative examples in the different training sets.

To identify the optimal ratios of examples from the largest categories in the training sets of the three SVMs, we introduced a second level of cross-validation (applied a stacked generalization procedure [40]). The criterion optimized was the MCC (in the case of M-SVM we used the sum of the three coefficients). In these procedures, sampling without replacement was used to select a number of examples from the two larger sets ranging from the number of examples of the smallest category to its maximum possible value (i.e., all examples of the large sets being retained). For each SVM and each value of the ratio, five different sets of negative examples were sampled. It appeared that the prediction accuracy was systematically increasing with the size of the training set. As a consequence, no example was discarded in any of the training sets of the five-fold cross-validation.

Table 2 presents the confusion matrix resulting from this procedure. The overall recognition rate exceeds 96.3%, with Matthews' correlation coefficients equal to 0.94 (signal peptide possessing proteins), 0.67 (type II signal anchor proteins), 0.82 (chloroplast targeted proteins), 0.83 (mitochondrion targeted proteins) and 0.95 (nuclear/cytosolic proteins).

**Table 2:** Prediction accuracy of HECTAR.

	**Signal peptide**	**Type II signal anchor**	**Chloroplast**	**Mitochondrion**	**No target peptide**
Signal peptide	428	1	5	1	6
Type II signal anchor	2	8	0	0	1
Chloroplast	10	0	43	0	2
Mitochondrion	8	1	1	103	15
No target peptide	6	3	0	14	1400

Confusion matrix of HECTAR predictions obtained by five-fold cross-validation. Each line represents one specific category of subcellular targeting. The columns indicate the categories of subcellular targeting predicted by HECTAR.

### Additional testing of HECTAR using proteins with known subcellular localisations

We established an additional set of proteins whose experimentally-determined subcellular localisations corresponded to one of the five categories predicted by HECTAR. This data set included one mitochondrion targeted protein, the TIM50 subunit protein from *Phytophthora infestans*, and a number of proteins of the cryptophyte *Guillardia theta*. Cryptophytes are a sister group to the heterokonts, and they are also believed to be derived from a secondary endosymbiosis event involving a red alga [21,22]. Like heterokonts, cryptophytes possess complex chloroplasts and chloroplast targeting is mediated by a bipartite target peptide consisting of an N-terminal signal peptide followed by a chloroplast transit peptide. Chryptophyte chloroplast targeting peptides possess an ASAFAP-like motif (AXAF), with a highly conserved phenylalanine, at the signal peptide cleavage site [41]. Ten cryptophyte proteins that have been shown experimentally to be targeted to different subcellular localisations were analysed by HECTAR. These included one cytosolic, three secreted proteins and two categories of chloroplast targeted proteins. The first were proteins that are targeted to the interior of the cryptophyte chloroplast (three proteins) whereas the second corresponded to proteins that are targeted to the periplastid space between the second and third (outermost) chloroplast membranes (three proteins) [22,42]. Proteins targeted to the periplastid space possess a bipartite target peptide but they do not have a conserved phenylalanine after the signal peptide cleavage site. It has been demonstrated that this phenylalanine residue is essential for a protein to be transported further into the chloroplast [41]. Table 3 shows that HECTAR successfully identified the presence of signal peptides in the proteins that enter the secretory pathway. It also correctly predicted the subcellular localisation of the mitochondrial proteins, the cytosolic protein and the proteins that are targeted into the interior of the cryptophyte chloroplast. The proteins that are targeted to the periplastid space of the cryptophyte chloroplast were predicted to be either secreted or chloroplastic. This is not surprising because HECTAR has not been designed to identify this class of targeting sequence. This analysis confirmed the reliability of the predictions produced by HECTAR and demonstrated that it can also accurately predict the subcellular localisation of proteins from organisms such as the cryptophytes that belong to groups other than the heterokonts but possess complex plastids derived from a secondary endosymbiosis event.

**Table 3:** Test of HECTAR with additional experimentally validated proteins.

**AccNr**	**Gene name**	**Species**	**HECTAR prediction**	**Evidenced localisation**
AY751575	TIM50	*Phytophthora infestans*	mitochondrion	mitochondrion
AJ937545	cycb	*Guillardia theta*	signal peptide	secreted
AJ937544	cath	*G. theta*	signal peptide	secreted
AJ937546	psi	*G. theta*	signal peptide	secreted
AJ937535	mpheS	*G. theta*	chloroplast	chloroplast
AF268324	LHCC13	*G. theta*	chloroplast	chloroplast
U40032	GapC1	*G. theta*	chloroplast	chloroplast
AJ937542	iddi	*G. theta*	signal peptide	periplastid space
AJ937543	hemE	*G. theta*	chloroplast	periplastid space
AJ784213	gbss	*G. theta*	signal peptide	periplastid space
U39873	GapC2	*G. theta*	no target peptide	cytosol

HECTAR was applied on eleven proteins with known subcellular localisation.

### Analysis of putative Fucus distichus secreted proteins using HECTAR

Belanger et al. [43] used a yeast signal sequence trap (SST) screen to identify secreted proteins potentially involved in asymmetric zygote cell growth in the brown alga *Fucus distichus*. The putative secreted proteins identified in this study included several probable chloroplast proteins such as fucoxanthin a/c-binding binding proteins (FCP) and the authors suggested that these may have been *bona fide *chloroplast proteins that were recognised by the yeast secretion machinery as secreted proteins due to their N-terminal signal peptide. To test whether HECTAR could distinguish between the secreted and the chloroplast proteins in this data set, we selected the protein sequences that were at least 100 residues long and applied a redundancy reduction (for details see Methods). The remaining 47 *F. distichus *putative secreted proteins were analysed by HECTAR. Of the 47 proteins, 45 were predicted to possess a N-terminal signal peptide (see Table 4). No target peptide was found in two of the proteins (BU037984 and BU038066). The SST procedure has been shown to select a small percentage of non-secreted proteins as false positives [44]. This occurs because these proteins possess a short region that shares some similarity with signal peptides at their N-terminal end. BU037984 and BU038066 may belong to this category. The analysis by HECTAR suggested that nine of the 45 proteins with a signal peptide also possessed a chloroplast transit peptide, indicating that these proteins are targeted to the chloroplast. Comparison with the Genbank non-redundant protein database (NR) using BlastP allowed putative functions to be assigned to 23 of the 47 Fucus proteins (see Table 4). BlastP searches with the other 24 proteins either returned matches with proteins of unknown function or did not find any matches in the database. Additional searches were carried out against published heterokont genomes, and using the Pfam database, but no additional functional information was obtained for these proteins (data not shown). The putative functions of the 23 proteins which matched proteins with functional information in the database were consistent with the subcellular localisations predicted by HECTAR.

**Table 4:** HECTAR analysis of putative secreted proteins from Fucus distichus.

**AccNr**	**HECTAR**	**BlastP against NR**	**BlastP match AccNr**	**E-value**
BU037999	signal peptide	no hits found		
BU038005	signal peptide	no hits found		
BU038011	signal peptide	no hits found		
BU038014	signal peptide	Archae adhesin-like	YP_001273569	6.00E-005
BU038016	signal peptide	heat shock cognate 70	ABH09735	2.00E-030
BU038019	signal peptide	Bacterial dipeptidase	YP_001358201	7.00E-012
BU038023	signal peptide	xylosyltransferase	XP_001658334	8.00E-010
BU038035	signal peptide	Bact. pentapeptide repeat containing	YP_001068500	0.014
BU038038	signal peptide	copper radical oxidase	ABD61575	3.00E-012
BU038040	signal peptide	Bact. cyclic nucleotide-binding domain	YP_527950	2.00E-003
BU038041	signal peptide	predicted protein	XP_001769436	4.00E-005
BU038044	signal peptide	no hits found		
BU038047	signal peptide	no hits found		
BU038050	signal peptide	no hits found		
BU038056	signal peptide	cysteine protease	BAD29957	4.00E-024
BU038058	signal peptide	no hits found		
BU038063	signal peptide	no hits found		
BU038071	signal peptide	no hits found		
BU038074	signal peptide	no hits found		
BU038076	signal peptide	no hits found		
BU038079	signal peptide	no hits found		
BU038082	signal peptide	no hits found		
BU038085	signal peptide	high CO2 inducible periplasmic	AAW79380	5.00E-004
BU038087	signal peptide	no hits found		
BU038091	signal peptide	mannuronan C-5-epimerase	CAD42950	0.001
BU038093	signal peptide	no hits found		
BU038094	signal peptide	Bact. hypothetical protein	YP_525510	0.004
BU038100	signal peptide	predicted protein	XP_001700285	5.00E-016
BU038108	signal peptide	no hits found		
BU038114	signal peptide	glutathione peroxidase	ABN46985	1.00E-024
BU038121	signal peptide	Bact. catalase	YP_458982	2.00E-015
BU038126	signal peptide	no hits found		
BU038127	signal peptide	no hits found		
BU038130	signal peptide	cysteine protease	ABQ10203	1.00E-004
BU038146	signal peptide	no hits found		
BU038148	signal peptide	FK506-binding	EDS41102	7.00E-007
BU037981	chloroplast	light harvesting	AAG13008	8.00E-062
BU037991	chloroplast	heat shock protein 70	AAM94003	1.00E-010
BU038012	chloroplast	chloroplast LI818 protein	ABD58893	1.00E-010
BU038052	chloroplast	GAPDH precursor	AAQ13415	3.00E-103
BU038060	chloroplast	extrinsic protein in PSII	CAH25361	0.016
BU038064	chloroplast	chlorophyll a/b-binding	AAP79202	1.00E-018
BU038101	chloroplast	sirohydrochlorin ferrochelatase	NP_564562	4.00E-021
BU038123	chloroplast	no hits found		
BU038142	chloroplast	chloroplast light harvesting	ABA55527	6.00E-008
BU037984	no target peptide	no hits found		
BU038066	no target peptide	no hits found		

HECTAR was applied on putative secreted *Fucus distichus *proteins. The localisations predicted by HECTAR as well as the details of BlastP searches against the NCBI non-redundant protein database (NR) are listed. Only matches were taken into account with an e-value of less than 0.02.

### HECTAR^*SEC*^

**Figure 4:**
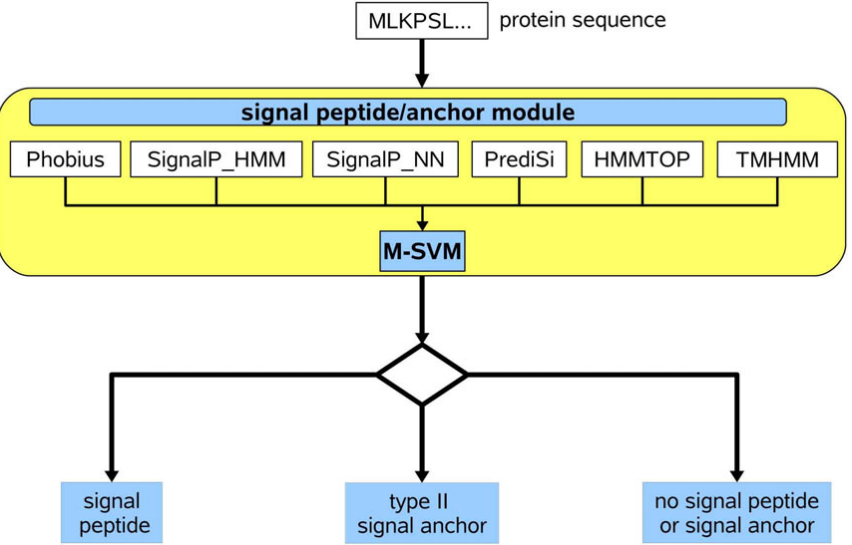
**Architecture of HECTAR^*SEC*^**. HECTAR^*SEC *^is a variant of HECTAR that is dedicated to identifying signal peptides and type II signal anchors in proteins from any eukaryotic organism. This method implements the HECTAR "signal peptide/anchor" module.

HECTAR has been designed to predict the subcellular localisation of heterokont proteins. As a result, it cannot be used to determine the subcellular localisation of proteins from green plants because their chloroplast targeting peptides consist of a single unit, the chloroplast transit peptide. However, because signal peptides and type II signal anchors have a similar composition in all eukaryotes [25,28] and HECTAR has been trained on target peptides from across the eukaryotic tree, the "signal peptide/anchor" module can be used to identify these two types of target peptides in a protein from any eukaryotic organism. We call this version of HECTAR, consisting of only the "signal peptide/anchor" module, HECTAR^*SEC *^(Fig. 4). A five-fold cross-validation of HECTAR^*SEC*^ showed that it predicted the presence of signal peptides and type II signal anchors with a high (98.4%) accuracy and that with MCCs of 0.96 (signal peptide), 0.67 (type II signal anchor) and 0.96 (no signal peptide or signal anchor) (for details see Table 5).

**Table 5:** Prediction accuracy of HECTAR^*SEC*^.

	**Signal peptide**	**Type II signal anchor**	**No signal peptide or anchor**
Signal peptide	486	1	9
Type II signal anchor	2	8	1
No signal peptide or anchor	15	4	1532

Confusion matrix of HECTAR^*SEC *^predictions obtained by five-fold cross-validation. Each line represents one specific category of subcellular targeting. The columns indicate the categories of subcellular targeting predicted by HECTAR^*SEC*^.

## Conclusion

To date, complete genome sequences have been published for three heterokonts: The diatom *Thalassiosira pseudonana *[45] and two oomycete plant pathogens [46]. In addition, genome sequencing has been completed or is nearing completion for several other heterokont species, including the diatoms *Phaeodactylum tricornutum*, *Fragilariopsis cylindrus *and *Pseudo-nitzschia*, the oomycetes *Phytophthora infestans *and *Phytophthora capsici*, the pelagophyte *Aureococcus anophagefferens*, the chrysophyte *Ochromonas danica *and the brown alga *Ectocarpus siliculosus*. This rapidly increasing availability of sequence data for the heterokonts brings with it a need for specialised bioinformatics tools to identify genes and to make predictions about the characteristics of the encoded proteins. HECTAR addresses one aspect of this problem, the prediction of the subcellular localisation of heterokont proteins. We have shown that HECTAR fulfills this function with high efficiency using cross-validation and by further tests with additional proteins from several species including the cryptophyte *Guillardia theta *and the brown alga *Fucus distichus*.

In its present form, HECTAR can discriminate between four types of target peptides. However, because of its modular architecture, it could easily be adapted in the future to identify additional types of target peptides. These could include sequences that direct proteins to more precisely defined subcellular compartments (such as the signals that allow targeting to the chloroplast thylakoid membrane or the mitochondrial matrix, for example) or regions within the protein sequence such as membrane-spanning domains or nuclear localisation signals.

## Methods

### Data sets of proteins with experimentally verified subcellular localisations

The protein sequences that were used to train and test HECTAR were obtained either from Swiss-Prot or by searching the scientific literature. The latter method was particularly important for identifying heterokont proteins because of the small number of sequences that have been analysed experimentally for this group of organisms. The Swiss-Prot database (release 54.3) was searched with the Sequence Retrieval System (SRS version 7.1.3.2) at the EBI webpage for entries with the comment type (CC) "subcellular localisation". Proteins that had been marked as lacking their terminal end (non_ter) or the initial methionine at the N-terminus (init_met) or proteins containing non-consecutive residues within the sequence (non_cons) were excluded. We also implemented our own parsers in Perl to remove any protein that had uncertainties in the protein sequence (i.e., containing the residues X, B or Z), the note "CONFLICT" in the feature table entry, "By similarity", "Probable" or "Potential" in the "CC SUBCELLULAR LOCATION" or in the target peptide description of the feature table (FtKey) or multiple possibilities for subcellular localisation. In cases where the subcellular localisation did not agree with the target peptide annotation in the Swiss-Prot feature table entries, the proteins were also removed. Using this general approach, mitochondrial proteins were retrieved by searching for the subcellular localisation "mitochondrion" (CC) and for the feature table entries "TRANSIT" (FtKey) and "mitochondrion" (FtDescription). Nuclear/cytosolic proteins were recovered by searching for "nucleus" or "cytoplasm" in the CC field. Secreted proteins were identified by searching for the feature table key "SIGNAL". After removal of non-valid proteins as described above, we retained 167 mitochondrial targeted, 2330 nuclear/cytosolic and 977 secreted proteins. Searches of the scientific literature concentrated on the identification of two classes of protein: proteins with a type II signal anchor and chloroplast targeted proteins from heterokonts. Proteins with type II signal anchors were only accepted if they had been shown experimentally to be anchored in the cell membrane. We also verified that the N-terminus of the proteins had been shown to be orientated towards the cytosol (N_*in*_), and the C-terminus to be located either in the lumen of a subcellular compartment (of the secretory pathway) or on the outside of the cell (C_*out*_). For the latter, glycosolation of the C-terminal part of the protein was accepted as evidence of a luminal/extracellular location for this part of the protein. Proteins with more than one transmembrane spanning region were eliminated. Eleven type II signal anchor proteins were found in this way.

To identify experimentally verified heterokont chloroplast targeted proteins, we searched for evidence based either on the uptake of GFP or red fluorescent protein (RFP) fusion proteins into heterokont chloroplasts, on the import of proteins into canine microsomes (to validate the N-terminal signal peptide) or on screening of libraries with antibodies against FCP. For some of the heterokont chloroplast targeted proteins, conservation of targeting sequences in a multiple alignment was also accepted as proof. A collection of 62 manually curated chloroplast proteins from the diatoms *Thalassiosira pseudonana *and *Phaeodactylum tricornutum *constituted the major part of the heterokont chloroplast data set. These sequences were kindly provided by Peter Kroth (University of Konstanz, Germany). Together with additional chloroplast targeted proteins from diatoms, brown algae and raphidophytes, the heterokont chloroplast protein data set totaled 72 proteins.

We implemented a redundancy reduction pipeline to remove redundant data from the above data sets. For this, ClustalW was modified so that it provided a pairwise distance matrix. This information was then fed to an in house implementation of the Hobohm2 algorithm [47]. The redundancy reduction was applied individually to each of the data sets corresponding to the five categories of subcellular targeting. A protein sequence was defined as non-redundant if its 100 N-terminal residues showed a sequence identity of less than 35% with the other proteins of the same reference set. After redundant sequences had been removed, the final data sets included 128 mitochondrial targeted proteins, 1423 nuclear/cytosolic proteins, 441 secreted proteins, 11 type II signal anchor proteins and 55 heterokont chloroplast targeted proteins.

### ASAFAP motif search

To develop a search procedure for the conserved motif identified in [24], we applied the signal peptide prediction algorithms (SignalP_NN, SignalP_HMM, Phobius and PrediSi) to the 55 proteins of the heterokont chloroplast data set and aligned these proteins at their predicted signal peptide cleavage sites. In rare cases where the prediction methods did not agree on the cleavage site position for an individual protein, we searched the sequence surrounding the predicted cleavage sites for traces of the ASAFAP motif and the alignment was then manually improved with respect to the conserved motif. The Shannon entropy [48]*S*_*obs*,*i *_was evaluated for each of the six residues neighbouring the signal peptide cleavage site (positions -3 to +3):

$$S_{obs,i}=-\sum_{j=1}^{20}f(x_{i}=a_{j})\log_{2}(f(x_{i}=a_{j})),$$

where {*a*_*j*_: 1 ≤ *j *≤ 20} is the set of the 20 natural amino acids and *f*(*x*_*i *_= *a*_*j*_) is the frequency of amino acid *a*_*j *_at position *i *within the ASAFAP motif with *i *∈ {-3, -2, -1, +1, +2, +3}.

By Schneider [49] the information content *R*_*seq*,*i *_which describes the conservation of the residue at position *i *of a protein sequence is:

*R*_*seq*,*i *_= *S*_*max *_- *S*_*obs*,*i *_= log_2 _(20) - *S*_*obs*,*i*_,

where *S*_*max *_is the maximum possible entropy.

To search for the ASAFAP motif, HECTAR scans from the N-terminal end of a protein sequence as far as the 20 first residues after the predicted signal peptide cleavage site using a six-residue sliding window. A score is computed for each window. This score represents the similarity of each window content to the consensus motif. For this the amino acid frequencies and the information content for each position of the ASAFAP motif retrieved in the above mentioned procedure are taken into account. It is given by:

$$score(w_{k})=\sum_{i=k}^{k+5}f(x_{i})R_{seq,i},$$

where *w*_*k *_is the content of the six-residue window starting at position *k *of the protein sequence. The best score for a protein is then transmitted to the chloroplast targeting module of HECTAR where it contributes to the detection of putative chloroplast targeted proteins.

### SVM classifiers

We have seen that HECTAR uses three SVMs as combiners: a M-SVM and two bi-class SVMs. We used our software, which implements the M-SVM of Weston and Watkins and is dedicated to very large data sets [37], to develop all three machines. This software is available at the following address: . This approach could be used since the M-SVM is identical to the bi-class SVM when applied to compute dichotomies. The kernels of the M-SVM ("signal peptide/anchor" module) and the bi-class SVM of the "mitochondrion targeted" module are RBF (Gaussian), whereas the kernel of the bi-class SVM of the "chloroplast targeted" module is linear. As a consequence, for the SVMs of the "signal peptide/anchor" module and the "mitochondrion targeted" module, model selection consisted of choosing the bandwidth of the kernel and the value of the *soft margin parameter C*, whereas for the "chloroplast targeted" module SVM, only the value of *C *had to be set. To peform this task, we studied the way guaranteed risks varied as a function of the values of these hyperparameters. The optimization procedure corresponding to *C *made use of the algorithm proposed in [50] to fit the entire regularization path. This allowed us to spare cpu time. In the bi-class case, the guaranteed risk used was the standard bound on the expected risk of kernel machines involving their Rademacher complexity (see [51], Section 3 for details). For the M-SVM, we used the multi-class extension of this bound established in Chapter 2 of [52]. Bounds were used for model selection in place of an additional cross-validation procedure for two reasons. The first was to keep as many examples as possible for training, given the fact that the size of some sets, such as the type II signal anchor proteins, is rather small. The second reason was to avoid complexifying the cross-validation procedure implemented to assess the generalization performance of HECTAR.

## Authors' contributions

BG, YG and JMC wrote the article. BG developed the HECTAR application. BG set up the experimental evidenced biological data sets which were used for training and testing HECTAR. BG also did the feature selections and the training and testing of each SVM. YG contributed his knowledge of SVMs and assisted at the SVM training and performance assessment. JMC directed the project. All authors read and approved the final manuscript.
